# Vertebral anomalies and VACTERL association in pontine tegmental cap dysplasia: a paediatric case report

**DOI:** 10.1093/bjrcr/uaad002

**Published:** 2023-12-13

**Authors:** Rachel Jane Klapper, Joshua Strobel, Chance Hebert, Maamannan Venkataraj, Tailong Xu, Octavio Arevalo Espejo

**Affiliations:** Department of Radiology, Louisiana State University Health Sciences Center-Shreveport, Shreveport, LA 71103, United States; Department of Radiology, Louisiana State University Health Sciences Center-Shreveport, Shreveport, LA 71103, United States; Louisiana State University Health Shreveport, School of Medicine, Shreveport, LA 71103, United States; The Department of Internal Medicine, Louisiana State University Health Sciences Center-Shreveport, Shreveport, LA 71103, United States; Department of Radiology, Louisiana State University Health Sciences Center-Shreveport, Shreveport, LA 71103, United States; Department of Radiology, Louisiana State University Health Sciences Center-Shreveport, Shreveport, LA 71103, United States

**Keywords:** vertebral anomalies, pontine tegmental cap dysplasia, paediatric, congenital vertebral defects

## Abstract

This case report highlights vertebral segmental anomalies and the fact that the child presented has a rare neurologic condition called pontine tegmental cap dysplasia. Additionally, this case aims to educate learners in developing a differential diagnosis for vertebral and cardiac anomalies such as VACTERL syndromes and common syndromes associated with butterfly vertebrae in children and adolescents.

## Introduction

Pontine tegmental cap dysplasia (PTCD) is a rare congenital malformation of the brainstem and hindbrain with diagnostic MRI findings of vermal hypoplasia, aplasia of the middle cerebellar peduncles, ectopic dorsal transverse pontine fibre projecting from the tegmentum into the fourth ventricle, and lateralized course of the superior cerebellar peduncle resulting in “molar tooth” appearance of the midbrain. On a midsagittal view, the pontine tegmentum resembles a cap or bulge projecting into the fourth ventricle, hence the name PTCD. PTCD is associated with multiple cranial nerve deficits as well as cardiac, gastrointestinal, genitourinary, and skeletal defects (Table 1).^1^ Of those skeletal defects, less than half of PTCD cases also report vertebral anomalies.^2^

**Table 1.** Clinical features seen in PTCD.

	Features seen in PTCD^a^	Our patient’s features
Neurological	Developmental delay Seizure Ataxia Cranial nerve deficits: Ocular movement abnormalities Trigeminal paresthesia Facial paralysis Impaired hearing Swallowing disorder	Patient had all neurological abnormalities except seizure
Physical features and abnormalities	Eye Ear Cardiovascular Pulmonary Gastrointestinal Genitourinary Skeletal Vertebral Rib Limb	Patient had all physical abnormalities except pulmonary, gastrointestinal, and genitourinary

Abbreviation: PTCD, pontine tegmental cap dysplasia.

a Adapted from Chong et al.^2^

## Case presentation

**Figure 1.**
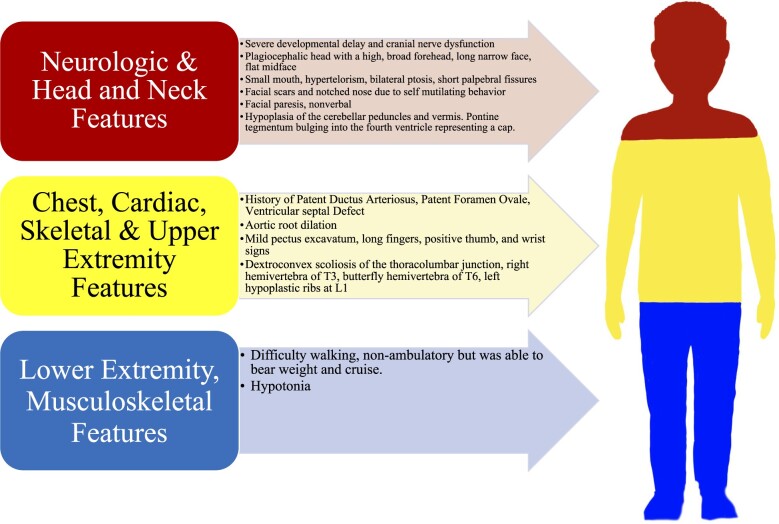
Diagram representing clinical and physical features of the 14-year-old male with PTCD presented in this case report including neurologic and head and neck features, chest, cardiac, skeletal, and upper extremity features, lower extremity, and musculoskeletal features. PTCD, pontine tegmental cap dysplasia.

A 14-year-old male with a history of dysmorphic features, severe developmental delay, cranial nerve dysfunction, and hindbrain malformation presented with difficulty walking. On exam, the patient had a normal-sized-plagiocephalic head with a high, broad forehead, long, narrow face, flat midface, small mouth, hypertelorism, bilateral ptosis, and short palpebral fissures. Facial scars and a notched nose due to self-mutilating behaviour were also present. Additionally, he had mild pectus excavatum, long fingers, positive thumb, and wrist signs. Neurologically, he had facial paresis, hypotonia, was nonverbal, and nonambulatory but was able to bear weight and cruise (Figure 1).

**Figure 2.**
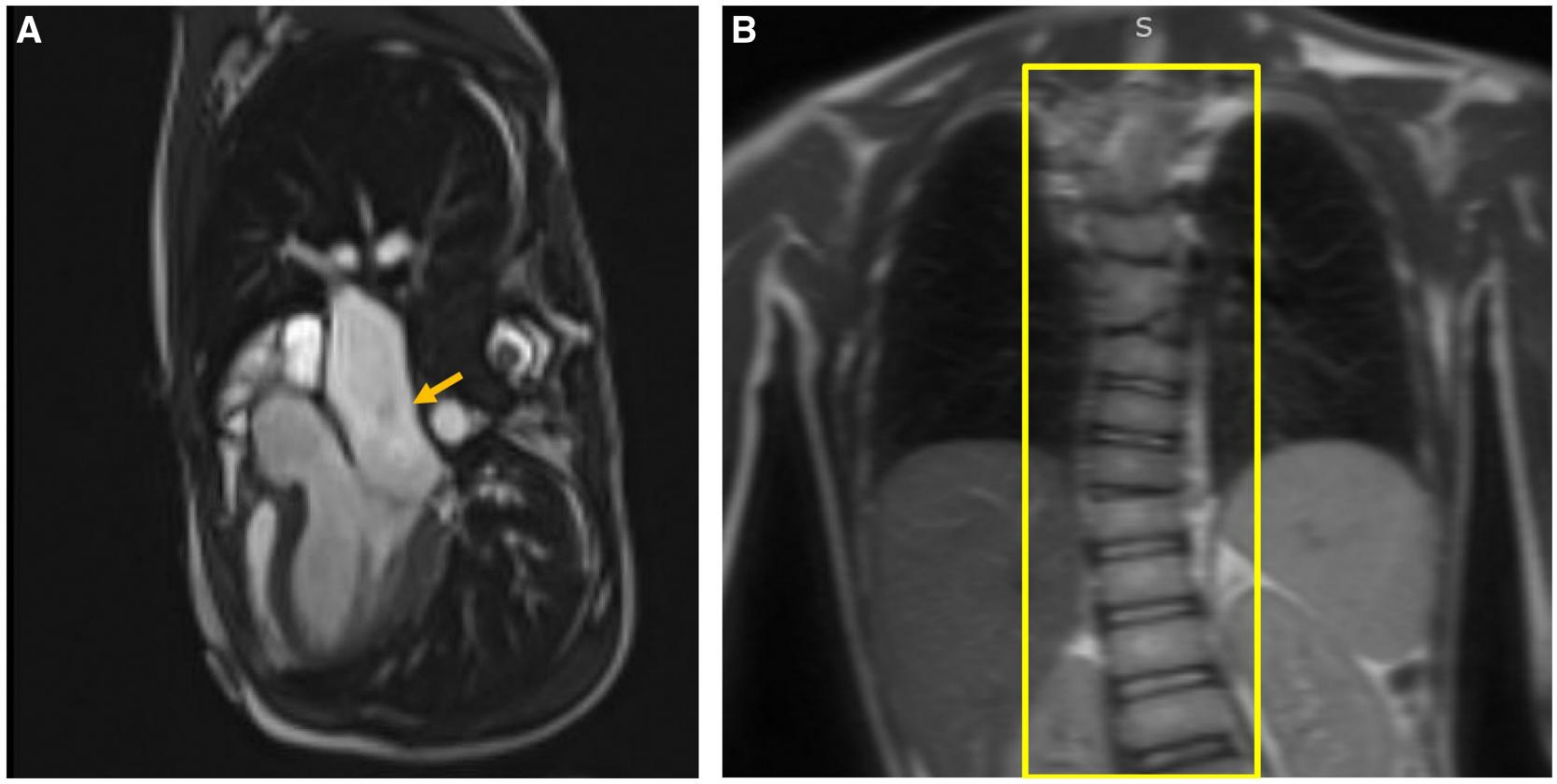
A, MRI Cardiac Morphology with and without contrast 3-chamber long axis view, yellow arrow shows aortic root dilation. B, Cardiac MRI coronal thorax view, yellow box displays dextroconvex scoliosis of the thoracolumbar junction, and multilevel malformations of the thoracic spine segmentation.

The patient was initially sent for cardiac evaluation shortly after birth due to failure to thrive and the presence of dysmorphic features. Cardiac history included Patent Ductus Arteriosus, Patent Foramen Ovale, and Ventricular Septal Defect, all of which spontaneously closed. MRI Cardiac Morphology was performed and revealed moderately enlarged aortic root. Left ventricular ejection fraction (LVEF) was slightly reduced at 48%; however, imaging was performed while the patient was under anaesthesia. Normal right ventriular ejection fraction (RVEF) 54% was seen (Figure 2A). Possible aortopathy related to connective tissue disease was found such as Marfans so he was sent to have genetic testing for the same which was negative.

**Figure 3.**
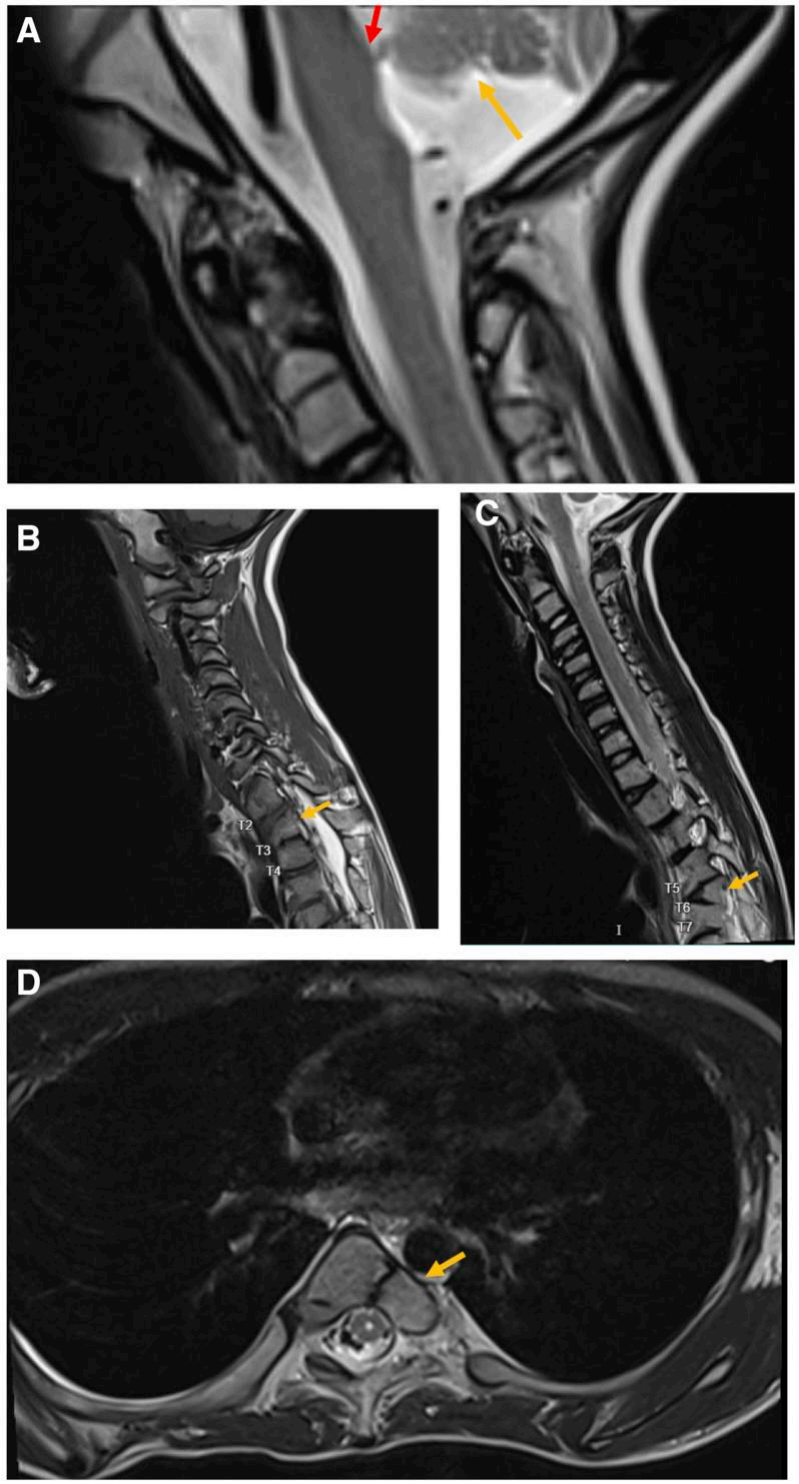
A-D, MRI Spine Cervical-Thoracic-Lumbar without contrast sagittal view T1 weighted. A, The inferior arrow displays partially visualized hypoplasia of the cerebellar peduncles and vermis. The superior arrow demonstrates the pontine tegmentum bulging into the fourth ventricle representing a cap. B, The arrow shows right hemivertebra of T3. C, The arrow shows the butterfly hemivertebra of T6. D, MRI Spine Cervical-Thoracic-Lumbar without contrast axial view T2 weighted. The arrow shows the axial plane through the hemivertebra.

MRI Spine Cervical-Thoracic-Lumbar without contrast was performed. The findings included evidence of hindbrain malformations including hypoplasia of the cerebellar peduncles and vermis (Figure 3A). In addition, dextroconvex scoliosis of the thoracolumbar junction, secondary to multilevel malformations of the thoracic spine segmentation was also seen (Figure 2B). There was a right hemivertebra of T3, butterfly hemivertebra of T6, and left hypoplastic ribs at L1 (Figure 3B-D).

## Discussion

Vertebral anomalies are also known to be associated with other malformations, syndromes, and genetic abnormalities. Differential diagnosis for a child or adolescent with vertebral anomalies includes Alagille syndrome, Jarcho-Levin syndrome, Joubert syndrome, Crouzon syndrome, Pfeiffer’s syndrome, and VACTERL syndromes. Studies have found that vertebral defects are commonly seen with cardiac, urinary, and digestive defects.^3^ VACTERL syndrome is when 3 or more anomalies including vertebral, anorectal, cardiac, tracheoesophageal fistula, renal anomalies, and limb defects are seen together.^3^ VACTERL syndrome incidence occurs approximately 1 in 10 000 to 1 in 40 000 live births.^4^ Vertebral anomalies are also associated with Kippel-Feil syndrome, Goldenhar syndrome, Juberg-Hayward syndrome, Aicardi syndrome, and Gorlin syndrome. Common vertebral anomalies involve the vertebral arch, as in spina bifida, or the vertebral body. Hemivertebra, block vertebra, and butterfly vertebra are all examples of vertebral body anomalies.

A butterfly vertebra is a sagittal defect in the vertebral body which is caused by failure of the lateral chondrification centres to fuse during embryogenesis, in contrast to a hemivertebrae that has complete developmental failure of one of the lateral chondrification centres.^5^ Persistent notochordal tissue remains in between or above and below the lateral halves of the affected vertebral body.^5^ Butterfly vertebrae are most associated with Alagille syndrome, Jarcho-Levin syndrome, Crouzon syndrome, Pfeiffer’s syndrome, and VACTERL syndromes.^4^^,^^5^ The vertebral body failure of fusion during embryogenesis represents a mesodermal error explaining why other organ systems with mesoderm origins are associated with butterfly vertebrae.^5^ While vertebral body anomalies have been recognized in these disorders, limited research has been done on the association between vertebral anomalies and PTCD. Only a few of the PTCD case reports have noted butterfly vertebrae and hemivertebrae, focusing more on neurologic findings.^6^^,^^7^

Our patient had aortic root dilation with a normal-sized ascending aorta. On reviewing other case reports on PTCD, multiple cardiac anomalies have been described such as VSD, atrial septal defects, aortic arch hypoplasia, and Tetralogy of Fallot.^6^ It is reported that cardiovascular anomalies are seen in 36% of patients with PTCD.^2^

## Conclusions

PTCD was first described in 2007 by Barth et al.^8^ As a recently characterized and rare disorder, more research is needed to fully understand the pathogenesis of the syndrome. A brief review of the literature demonstrates the rarity of PTCD with only 26 case report articles written since 2007-2023. The child presented in this case was diagnosed with PTCD and was found to have segmental vertebral body anomalies. This report focuses on a rare case where the patient has both hemivertebra and butterfly vertebra vertebral body anomalies with a known diagnosis of PTCD. The case provides motivation to further understand the cardiological and musculoskeletal implications of PTCD to assist physicians in caring for these patients in the future.

## Learning points

- Recognize vertebral anomalies such as hemivertebrae and butterfly hemivertebrae on MRI.
- Develop a differential for a child or adolescent with vertebral anomalies such as Alagille syndrome, Jarcho-Levin syndrome, Joubert syndrome, Crouzon syndrome, Pfeiffer’s syndrome, and VACTERL syndromes.
- Shed light on the rare neurologic condition pontine tegmental cap dysplasia and the common radiologic and clinical findings associated with the diagnosis.
